# In the national epidemiological bulletins – a selection from current issues

**DOI:** 10.2807/1560-7917.ES.2016.21.48.30411

**Published:** 2016-12-01

**Authors:** Eurosurveillance editorial team

**Affiliations:** 1 European Centre for Disease Prevention and Control (ECDC), Stockholm, Sweden

**Keywords:** public health

**Healthcare-associated infections and antibiotic resistance**
Colistin resistance in Gram-negative bacteria - the situation in Germany
Epidemiologisches Bulletin 46, 2016
21 November 2016, Germany
https://www.rki.de/DE/Content/Infekt/EpidBull/Archiv/2016/Ausgaben/46_16.pdf?__blob=publicationFile

**Respiratory diseases**
Online survey on influenza vaccination among hospital staff
Epidemiologisches Bulletin 47, 2016
28 November 2016, Germany
http://www.rki.de/DE/Content/Infekt/EpidBull/Archiv/2016/Ausgaben/47_16.pdf?__blob=publicationFile
Surveillance of *Streptococcus pneumoniae* causing bacteraemia in England, Wales and Northern Ireland: 2015
Health Protection Report; 10(40)
18 November 2016, United Kingdom
https://www.gov.uk/government/uploads/system/uploads/attachment_data/file/571420/hpr4016.pdf

**Vaccine-preventable diseases**
Epidemic occurrence of whooping cough
EPI-NEWS 46, 2016
16 November 2016, Denmark
http://www.ssi.dk/English/News/EPI-NEWS/2016/No%2046%20-%202016.aspx
The first year with statutory registration of vaccinations in the Danish Vaccination Register
EPI-NEWS 45, 2016
9 November 2016, Denmark
http://www.ssi.dk/English/News/EPI-NEWS/2016/No%2045%20-%202016.aspx

**Sexually transmitted diseases**
Special edition: World AIDS Day, December 1, 2016
Bulletin épidémiologique hebdomadaire, 41-42
29 November 2016, France
http://invs.santepubliquefrance.fr/beh/2016/41-42/pdf/2016_41-42.pdf
HIV infection and AIDS: Quarterly report to 30 September 2016
HPS Weekly Report, 2016;50(48)
28 November 2016, Scotland
http://www.hps.scot.nhs.uk/documents/ewr/pdf2016/1648.pdf
Estimated number of new HIV infections and estimated total number of people with HIV in Germany
Epidemiologisches Bulletin 45, 2016
14 November 2016, Germany
https://www.rki.de/DE/Content/Infekt/EpidBull/Archiv/2016/Ausgaben/45_16.pdf?__blob=publicationFile

**Zoonoses and vector-borne diseases**
Selected parasite infections detected using the PCR method
EPI-NEWS 44, 2016
2 November 2016, Denmark
http://www.ssi.dk/English/News/EPI-NEWS/2016/No%2044%20-%202016.aspx

**Other**
Special edition: Asylum seekers and infectious diseases
Infectieziekten Bulletin 2016; 27(9)
November 2016, the Netherlands
http://www.rivm.nl/Documenten_en_publicaties/Algemeen_Actueel/Uitgaven/Infectieziekten_Bulletin/Jaargang_27_2016/November_2016/Inhoud_november_2016

